# Supplementary material for: Modeling glioblastoma heterogeneity as a dynamic network of cell states
Source: Mol Syst Biol. 2021 Sep 16;17(9):e10105. doi: 10.15252/msb.202010105 (PMC8444284; doi:10.15252/msb.202010105)
Supplement: Supplementary file 6 — Source Data for Figure 5 [file MSB-17-e10105-s004.zip › Figure5A_sourcedata/GSEA_3071/hallmarks_stateIV.GseaPreranked.1623742551702/HALLMARK_PANCREAS_BETA_CELLS.html]

Details for gene set HALLMARK\_PANCREAS\_BETA\_CELLS[GSEA]

|  || Dataset | state53071 |
| Phenotype | NoPhenotypeAvailable |
| Upregulated in class | na\_pos |
| GeneSet | HALLMARK\_PANCREAS\_BETA\_CELLS |
| Enrichment Score (ES) | 0.48547527 |
| Normalized Enrichment Score (NES) | 1.6457347 |
| Nominal p-value | 0.03586498 |
| FDR q-value | 0.046154104 |
| FWER p-Value | 0.255 |
Table: GSEA Results Summary

  

Fig 1: Enrichment plot: HALLMARK\_PANCREAS\_BETA\_CELLS      
 Profile of the Running ES Score & Positions of GeneSet Members on the Rank Ordered List

  

| PROBE | GENE SYMBOL | GENE\_TITLE | RANK IN GENE LIST | RANK METRIC SCORE | RUNNING ES | CORE ENRICHMENT || 1 | DCX |  |  | 4 | 2.752 | 0.2207 | Yes |
| 2 | INSM1 |  |  | 46 | 1.869 | 0.3634 | Yes |
| 3 | LMO2 |  |  | 272 | 1.099 | 0.4092 | Yes |
| 4 | FOXO1 |  |  | 663 | 0.679 | 0.3899 | Yes |
| 5 | PCSK2 |  |  | 694 | 0.663 | 0.4376 | Yes |
| 6 | PAK3 |  |  | 1046 | 0.506 | 0.4118 | Yes |
| 7 | NEUROD1 |  |  | 1180 | 0.471 | 0.4245 | Yes |
| 8 | MAFB |  |  | 1248 | 0.452 | 0.4482 | Yes |
| 9 | PAX6 |  |  | 1275 | 0.445 | 0.4790 | Yes |
| 10 | AKT3 |  |  | 1417 | 0.412 | 0.4855 | Yes |
| 11 | SPCS1 |  |  | 1679 | 0.353 | 0.4644 | No |
| 12 | SRP9 |  |  | 2151 | 0.276 | 0.3974 | No |
| 13 | VDR |  |  | 3524 | -0.414 | 0.1706 | No |
| 14 | SEC11A |  |  | 4647 | -0.763 | 0.0193 | No |
| 15 | FOXA2 |  |  | 5092 | -1.272 | 0.0375 | No |
Table: GSEA details [plain text format]

  

Fig 2: HALLMARK\_PANCREAS\_BETA\_CELLS: Random ES distribution      
 Gene set null distribution of ES for **HALLMARK\_PANCREAS\_BETA\_CELLS**
